# Leptin Receptor Gene Gln223Arg Polymorphism Is Not Associated with Hypertension: A Preliminary Population-Based Cross-Sectional Study

**DOI:** 10.1155/2014/879037

**Published:** 2014-02-13

**Authors:** Geórgia das Graças Pena, Andre L. S. Guimarães, Rosângela R. Veloso, Tatiana C. Reis, Crizian S. Gomes, João F. R. Neto, Gustavo Velasquez-Melendez

**Affiliations:** ^1^Maternal-Child Nursing and Public Health Department of the Nursing School, Nursing School, Federal University of Minas Gerais, (UFMG), 30130-100 Belo Horizonte, MG, Brazil; ^2^Department of Dentistry, Program in Health Sciences, State University of Montes Claros (UNIMONTES), 39401-001Montes Claros, MG, Brazil

## Abstract

Hypertension is responsible for high morbidity and mortality as one of the most important cardiometabolic risk factors. The aim of the study was to investigate whether the Gln223Arg in the leptin receptor (LEPR) influences the prevalence of hypertension. A cross-sectional study was carried out in individuals aged ≥ 18 years. Polymorphism identification was performed using PCR-RFLP analysis. Participants with blood pressure ≥ 140/90 mmHg or medication use were considered hypertensive. Frequencies, means, cross-tabulations, and multivariate models were produced to study differences in hypertension prevalence by genotypes. The study includes 470 participants. The frequency of GG polymorphism variant was 10.43%, 46.81% AG, and 42.77% AA. The distribution of hypertension frequency by LEPR genotypes was the following: AA 43.8%, AG 40.4%, and GG 40.8%; there were no significant differences between groups. Comparative analysis which used multivariate Poisson regression adjusted by many potential confounders (age, sex, schooling, smoking, alcohol intake, obesity, and family history of parental obesity) did not modify this result. In this large sample of population-based study, the association of the LEPR Gln223Arg gene polymorphism with hypertension was not observed.

## 1. Introduction

Cardiometabolic outcomes are responsible for high mortality worldwide both in developed and developing societies [1, 2]. Among the top ten causes of death, two are cardiovascular diseases (CVD) and responsible for 12.8% of the deaths in the world [3].

Moreover, CVD are related to complex phenotypes with multifactorial etiology such as obesity, uncontrolled blood pressure, increased waist circumference, increased fractions of LDL and triglycerides, decreased HDL [2, 4–9], presence of metabolic syndrome [10–12], diabetes [13], and genetic polymorphisms [14]. These complex phenotypes contribute to the increase of the prevalence of CVD and increasing the costs in the public health [15].

Therefore, studies which investigate the association of these complex phenotypes, such as cardiometabolic risk factors, take on a definite importance in the context of public health and the search of different analysis tools such as evaluation of polymorphisms. These are interesting strategies to understand the contribution of the epidemiology of CVD better.

The mechanism of leptin signaling in the regulation of energy homeostasis in human metabolism [16] is considered of utmost importance. Besides the leptin gene receptor, polymorphism possibly intermeddles with the regulation of body weight, obesity, fat mass distribution, serum leptin levels, glucose homeostasis, and diabetes, among others [17–24].

The variants of the leptin receptor Gln223Arg gene (LEPR Gln223Arg) have been reported to associate with metabolic functions and adiposity in different studies, contribute to inadequate metabolism of the hormone, and affect the biological functions and leptin resistance [19, 21, 24, 25]. Thus, this polymorphism could be involved as a genetic risk factor in overweight and other cardiometabolic events [17, 20, 22–24]. Evidence of a significant effect of the Gln223Arg polymorphism on blood pressure regulation has been reported in some recent studies [21, 26, 27]. The aim of the study was to study the association of the LEPR Gln223Arg with prevalence of hypertension in an urban Brazilian population.

## 2. Methods

### 2.1. Study Population and Design

A cross-sectional population-based study was conducted with residents aged ≥18 years in Montes Claros, Minas Gerais, Brazil. Montes Claros now has about 361.915 inhabitants—95.1% of them in the urban municipality area [28].

### 2.2. Sample Design

This research proposes the sample size procedure for estimating the prevalence of the LEPR Gln223Arg. The sample size was based on the expected prevalence of 10% of the less frequent polymorphism with the standard deviation of 1.55 and variation coefficient of 15%. In the first stage, the database of census tracts (2010 Census, IBGE) [28] was used for drawing the primary sampling units. In the second stage, the address list for the purpose of drawing households and all individuals in the survey asked to participate in the study will be used.

### 2.3. Data Collection

An interview was conducted by answering a face-to-face survey questionnaire covering various aspects of their demographic (sex, age, skin color, marital status, and schooling) and lifestyle characteristics (physical activity, smoking habits, and alcohol consumption). At the conclusion of the interview, a clinical evaluation of participants was performed; this included weight, height, waist circumference, and blood pressure measurements, carried out threefold, by well-trained staff in keeping with standard procedures [29, 30].

Anthropometric variables were measured as suggested in the recommendations of the World Health Organization [30] and included all eligible participants in the selected households. For the adults, overweight was defined as BMI ≥ 25 kg/m^2^ and obese as BMI ≥ 30 kg/m^2^. Waist circumference (WC) was measured to the nearest millimeter, using a nonextendable measuring tape, and taken exactly halfway between the margin of the lowest rib and the iliac crest. The participants were in a standing position (accurate to 0.1 cm).

The capillary whole blood was obtained without fasting from a finger prick and was immediately analyzed with the use of a Performa (Roche Diagnostic Systems^©^, Nutley, NJ, USA) blood glucose analyzer. Participants were considered normal at less than 140 mg/dL or high when greater than or equal to 140 mg/dL.

Blood pressure was measured, using an ONRON HEM-742INT^©^ automatic BP monitor, in the sitting position, using the right upper arm and an appropriately sized cuff after a 5 min interval, according to The Seventh Report of the Joint National Committee on Prevention, Detection, Evaluation, and Treatment of High Blood Pressure [29]. Measurements were performed three times in the participant's right arm with a two-minute interval, after a rest period of at least five minutes. Hypertension was defined as a systolic blood pressure greater than or equal to 140 mmHg and/or a diastolic blood pressure greater than or equal to 90 mmHg or reported use of medication for hypertension control [29].

### 2.4. DNA Extraction and Genotyping

The collection of genomic DNA was obtained through an oral swab performed with a sterile plastic spatula. After gentle scraping of oral mucosa, the tip of the spatula was immersed in 2 mL sterile microtubes containing 1500 mL of Krebs buffer (NaCl 20%, KCl 2%, CaCl_2_, 2%, H_2_O 2%, MgSO_4_, KH_2_PO_4_, and C_6_H_12_O_6_) and stored at −20°C in the Laboratory of Health Research, Universidade Estadual de Montes Claros for future DNA extraction. DNA extraction was carried out by protocol described earlier [31, 32].

Gln223Arg (rs1137101) polymorphisms were assessed by PCR amplification and digestion. The primers used were 5′-ACCCTTTAAGCTGGGTGTCCCAAATAG-3′ and 5′-CAATATTTATGGGCTGAACTGACATT-3′ primers. For amplification and genotyping, 100 ng of DNA was amplified using primers specific to 10 pmol of primers: forward, 2.5 *μ*L, dNTP mix (25 mM of each), 2.5 *μ*L 10X PCR buffer, 1.25 *μ*L magnesium chloride (50 mM), and 2.5 units of Platinum Taq DNA polymerase (Invitrogen^©^ Life Technologies, Carlsbad, USA). The conditions for the PCR assay were denaturation at 95°C for 5 min followed by 35 cycles of denaturation at 95°C for 1 min, annealing at 57.4°C for 1 min, and extension at 72°C for 1 min, and a final extension at 72°C for 10 min. This primer pair produced a fragment of 330 bp. The 330 bp PCR product was digested using the M*spI* restriction endonuclease (Promega^©^, Madison, WI, USA). The substitution from A to G allele produce a single cut site yielding two bands of 293 and 37 bp.

### 2.5. Statistical Analyses

Studied population characteristics were presented by the absolute and relative frequencies of the demographic and life style variables stratified by sex. Statistical differences were evaluated by Pearson's chi-square test, and the significance level was set at 5% (*P* < 0.05). First, sample distributions of the sociodemographic variables (age, sex, schooling, smoking, alcohol intake, obesity and family history of parental obesity, family income, nutritional, and marital status) according to genotype Gln223arg polymorphism were estimated. Subsequently, associations between Gln223arg polymorphism and hypertension were explored with the use of crude prevalence ratios (PR) with 95% confidence intervals; a logistic regression model was used. Adjusted PR for potential confounders were obtained using a multivariable Poisson regression model including all variables. All analyses were conducted in Stata version 12.0 software, StataCorp^©^, Texas, USA.

### 2.6. Ethics Committee Approval

This study was approved by the Research Ethics Committee of Universidade Federal de Minas Gerais (UFMG), in accordance with National Health Council Resolution 196/96. All of the subjects who took part in the study were informed about the objectives of the research and their rights as participants, and then they were asked to sign a consent form.

## 3. Results

In the preliminary results, sample size was 470 participants, 34.2% (161) males and 65.7% (309) females. The mean and standard deviation of age with average age of total population was 44.72 ± 17.99 years. Selected demographic characteristics according to sex are shown in Table 1. The age group with the highest frequency was 18 to 29 years 25% (119) followed by age greater or equal to 60 years 21% (101). Frequencies of schooling equal to or greater than 9 years of education and income were similar between the sexes. There were no statistical differences in sociodemographic characteristics between sexes, except for the marital status. Regarding the married status (with spouse), the highest frequency was observed in men (65.8%) than women (45.3%; *P* < 0.001). For the lifestyle habits, the consumption of alcohol and tobacco was higher in the male group (*P* < 0.05).

Table 2 shows high prevalence of abdominal obesity among the women. Prevalence of nutritional status, hypertension, and glucose levels was similar between sexes. The genotypes of Gln223Arg polymorphism in the LEPR gene distribution were the following: GG polymorphism variant was 10.43% (*n* = 49), 46.81% AG (*n* = 220), and 42.77% AA (*n* = 201).

In the univariate analysis, carriers of genotype AA presented a slightly higher prevalence of hypertension (43.78%) when compared to those who have GG genotype (40.8%). The prevalence ratios were not significant (RP = 1.07; CI 95%: 0.73–1.55). The distribution of hypertension frequency by genotypes LEPR was the following by group: AA 43.8% (*n* = 88), AG 40.4% (*n* = 88), and GG 40.8% (*n* = 20); there were no significant differences between groups (*P* = 0.76).

Comparative analysis using multivariate Poisson regression adjusted by many potential confounders (age, sex, schooling, smoking, alcohol intake, waist circumference, glucose levels, and obesity and family history of parental obesity) did not modify this result. According to the multivariable Poisson regression model, genotypes of Gln223Arg polymorphism did not remain significantly associated with hypertension in various multivariate models (PR = 0.95; 95% CI: 0.70–1.27) after adjustment for age, sex, schooling, smoking, alcohol intake, waist circumference, and parental obesity history (Table 3).

## 4. Discussion

In this population-based study, the frequencies of LEPR Gln223Arg and the association between genotype frequencies and the mild levels of blood pressure were estimated. Thus by far, this is the first population-base study describing a genotype frequency of the LEPR Gln223Arg variant in healthy populations of Brazil. The genotype frequencies of three categories of polymorphism studied correspond to 10, 47, and 42% for GG, AG, and AA, respectively. This is rather dissimilar to recent studies carried out in healthy American multiethnic population (African, African-American, African-Caribbean, Caucasian, Asian, and other ethnic groups) in which the frequencies is diverse. For example, AA ranged from 13.4% in African-Caribean to 37.9% in Caucasian, while the GG ranged from 14.16% in Caucasian to 34.62% in Asian/other minority ethnic groups. However, the genotype frequencies founded are more similar with the Caucasians [33].

Several lines of evidence suggest that most of the polymorphisms associated with signaling impairment of leptin action in the central level would correspond to Gln223Arg polymorphism and in turn be associated with high circulating of leptin levels, and this may play a role in the pathophysiology of hypertension obese [20, 22]. The mechanism that explains the relationship between obesity and hypertension could be mediated by the increase of sympathetic nervous system activity.

According to recent studies, positive relationships between plasma leptin and 24-hour blood pressure levels have been shown. In a cross-sectional study in a population of 70 nondiabetic, normotensive, and obese women, serum leptin levels were directly related to 24-hour blood pressure levels aside BMI; this evinces that leptin levels could be a crucial parameter for determining the blood pressure level [34]. Obesity-induced hypertension could be secondary to insulin resistance and/or hyperinsulinemia [35]. In obese hypertensive subjects, changes in serum leptin levels correlated to changes in blood pressure levels after weight loss regardless of insulin resistance confirming the role of leptin in the pathophysiology of hypertension in obese patients [36].

The relationship between serum leptin levels was also shown in hypertensive participants. After adjustment for confounders, the authors concluded that free leptin surrogates are associated with masked hypertension in nonobese normoglycemic subjects. Following the same lines of evidence, in a study of 284 volunteers with various levels of waist to hip ratio, the differences in blood pressure by LEPR variants remained significant after adjusting for the influence of obesity and body fat distribution, as well as insulin and leptin [21].

In this study, no association between Gln223Arg polymorphism and hypertension is reported. This association remained nonsignificant after controlling for many potential confounders and measuring leptin levels that were not measured. It is known that casual blood pressure level does not account for the blood pressure circadian oscillation and this lack of precision in the measurements of blood pressure could explain the nonassociation between polymorphism and hypertension [37].

The results should be interpreted cautiously since the preliminary data results are about a complex sample and need weighing adjustments for different probabilities of selection of each participant.

In conclusion, this study does not provide evidence for a role of the LEPR gene Gln223Arg polymorphism in increasing the prevalence of mild hypertension in a cross-sectional population-based study. Thus, this study failed to provide evidence for a differential prevalence of mild hypertension by groups of the LEPR Q223R gene polymorphism.

## Figures and Tables

**Table 1:** Sociodemographic characteristics by sex. Montes Claros, Brazil, 2013.

Variable	Sex				Total		
	Male		Female				P value*
	n	%	*n*	%	n	%	
Age groups (years)							0.472
18–29	40	24.84	79	25.57	119	25.32	
30–39	30	18.63	52	16.83	82	17.45	
40–49	20	12.42	58	18.77	78	16.60	
50–59	34	21.12	56	18.12	90	19.15	
≥60	37	22.98	64	20.71	101	21.49	
Skin color							0.061
White	26	16.25	73	23.70	99	21.15	
Nonwhite	134	83.75	235	76.30	369	78.85	
Marital status							<0.001
With spouse	106	65.84	140	45.31	246	52.34	
Without spouse	55	34.16	169	54.69	224	47.66	
Education (years)							0.410
0–5	19	11.88	52	16.88	71	15.17	
6-7	68	42.50	118	38.31	186	39.74	
8–11	24	15.00	38	12.34	62	13.25	
≥12	49	30.62	100	32.47	149	31.84	
Income (minimum wages)							0.251
<2	25	15.53	44	14.29	69	14.71	
2-3	74	45.96	121	39.29	195	41.58	
≥4	62	38.51	143	46.42	205	43.71	
Physical activity							0.089
Yes	113	70.19	239	77.35	352	74.89	
Inactive	48	29.81	70	22.65	118	25.11	
Smoking							<0.001
Yes	32	20.00	16	5.23	48	10.30	
Ex-smoking	43	26.87	35	11.44	78	16.74	
No	85	53.13	255	83.33	340	72.96	
Alcohol intake							0.013
Yes	60	37.27	81	26.21	141	30.00	
No	101	62.73	228	73.79	329	70.00	

*P value < 0.05 for differences between male and female (chi-square test).

**Table 2:** Nutritional status, glucose levels, and blood pressure status by sex. Montes Claros, Brazil, 2013.

Variables	Sex				Total		
	Male		Female				*P* value*
	*n*	%	*n*	%	*n*	%	
Nutritional status							0.099
Underweight	08	4.97	13	4.23	21	4.49	
Normal weight	73	45.34	123	40.07	196	41.88	
Overweight	54	33.54	90	29.32	144	30.77	
Obese	26	16.15	81	26.38	107	22.86	
Abdominal obesity							<0.001
No	135	83.85	178	57.79	313	66.74	
Yes	26	16.15	130	42.21	156	33.26	
Hypertension							0.632
Yes	96	59.63	176	57.33	272	58.12	
No	65	40.37	131	42.67	196	41.88	
Glucose level							0.574
Normal (<140 mg/dL)	137	85.09	267	86.97	404	86.32	
High (≥140 mg/dL)	24	14.91	40	13.03	64	13.68	

*P < 0.05 for differences between male and female (chi-square test).

**Table 3:** Multivariate Poisson regression model (prevalence ratio and 95% CI) for hypertension, Montes Claros, Brazil, 2013.

Models	PR	95% CI	*P* value
Model 1			
Genotype GG*	1	—	—
Genotype AG	0.98	0.68–1.43	0.954
Genotype AA	1.07	0.73–1.55	0.712
Model 2 (adjust: age)			
Genotype AG	1.02	0.75–1.38	0.887
Genotype AA	0.98	0.72–1.33	0.919
Model 3 (adjust: age and sex)			
Genotype AG	1.00	0.75–1.38	0.895
Genotype AA	0.97	0.72–1.32	0.891
Model 4 (adjust: age, sex, and education)			
Genotype AG	1.02	0.75–1.39	0.868
Genotype AA	0.97	0.72–1.32	0.881
Model 5 (adjust: age, sex, education, and smoke)			
Genotype AG	1.04	0.75–1.43	0.801
Genotype AA	0.98	0.72–1.35	0.941
Model 6 (adjust: age, sex, education, smoke, and alcohol intake)			
Genotype AG	1.04	0.75–1.42	0.808
Genotype AA	0.98	0.72–1.35	0.938
Model 7 (adjust: age, sex, education, smoke, alcohol intake, and WC)			
Genotype AG	1.03	0.75–1.43	0.808
Genotype AA	0.99	0.72–1.35	0.938
Model 8 (adjust: age, sex, education, smoke, alcohol intake, WC, and parental obesity history)			
Genotype AG	1.02	0.75–1.38	0.862
Genotype AA	0.95	0.70–1.27	0.748

*GG genotype as reference group. WC: waist circumference.
